# Optimization of cabin seating arrangement strategies based on the Wells–Riley risk theory

**DOI:** 10.1371/journal.pone.0294345

**Published:** 2023-11-20

**Authors:** Yanxi Liu, Xuan Cheng, Dengzhao Tang, Xinyue Wang

**Affiliations:** Department of Airport, School of Transportation Science and Engineering, Civil Aviation University of China, Tianjin, China; Chulalongkorn University Faculty of Medicine, THAILAND

## Abstract

Civil aviation transport is an important source of global respiratory disease spread due to the closely-spaced environment. In order to reduce the probability of infection of passengers, an improved Wells-Riley model for cabin passenger risk assessment have been given in this work, the cabin ventilation and passenger nose and mouth orientation were considered. The model’s effectiveness has been verified with published data. Finally, how the load factor and use of an empty seat scheme are associated with the number of infected people was assessed. The results demonstrated that the number of infected people positively correlates with the passenger load factor, and the most suitable load factor can be determined by controlling the final number of infected people with the condition of the epidemic situation in the departure city. Additionally, infection risk was found to be lower among passengers in window seats than in those in aisle seats and middle seats, and keeping empty seats in the middle or aisle could reduce the cabin average probability of infection by up to 37.47%. Using the model developed here, airlines can determine the optimal load factor threshold and seating arrangement strategy to improve economic benefits and reduce the probability of passenger infection.

## 1 Introduction

Infectious respiratory diseases, such as tuberculosis, influenza, and aspergillosis, have spread globally in recent years, posing a serious threat to people’s health and economic development. Coronavirus disease 2019 (COVID-19), caused by severe acute respiratory syndrome coronavirus 2 (SARS-CoV-2), escalated into a global pandemic in just 4 months [1]. COVID-19 has caused massive loss of life and jeopardized human development (e.g., social and economic) [2].

Regardless of the manner of transmission, infectious respiratory disease spreading causes extensive harm and must be addressed [3]. In 2019, 4.54 billion passengers traveled by airplane, highlighting the scale of the aviation transportation industry. Traveling for long periods in densely occupied cabins of commercial passenger airplanes elevates infection risk. Therefore, civil aviation transport is an important route for the global spread of respiratory diseases. To prevent the spread of infectious diseases on commercial aircraft, the Centers for Disease Control and Prevention (CDC) recommended that once airline crew identify sick and potentially infectious passengers, separate the sick traveler from others by 6 feet or move adjacent passengers without compromising flight safety or exposing additional passengers [4]. Due to the incubation period of the COVID-19 virus, implementing the measures that are effective against common infectious diseases mentioned above may present challenges.

Therefore, to reduce contact between individuals and the virus, the airlines to block middle seats to ensure each passenger had an empty seat between them. Due to the COVID-19 pandemic, there has been a significant decrease in global passenger numbers, leading to massive economic losses in the aviation industry [5]. Further affecting airline revenue, the Civil Aviation Administration (CAA) suspended airlines for one week if more than 5 passengers on the same route received positive nucleic acid tests. This policy caused thousands of flights and crew members to be grounded [6]. In addition, in-flight infections cause substantial harm to the physical and mental health of passengers. Therefore, there is an urgent need to study airline seating arrangement plans to optimize epidemic prevention policies, improve the passenger load factor, and effectively reduce passenger infection risk.

Limited research has been conducted on aircraft seating arrangement plans. Using bacteriophage MS2 as a surrogate for airborne SARS-CoV-2, the National Institute for Occupational Safety & Health (NIOSH) [7] modeled the relationship between SARS-CoV-2 exposure and seating proximity and compared exposure levels in vacant middle seat scenarios (VMS) with those in full occupancy scenarios. Depending on the modeling approach, the vacant middle seat method reduced exposure by 23% to 57% compared to that observed in the full occupancy scenarios. Using tracer particle data from the U.S. Transportation Command (TRANSCOM), computational fluid dynamics (CFD) simulation data from Boeing, and NIOSH data, Bennett and Mahmoud et al. [8] estimated the reduction in virus exposure and infection in VMS with passengers wearing medical masks and found that in a 24-row cabin with vacant middle seats, reductions in virus exposure averaged 36% when there was one infectious passenger present. More research has been conducted on high-speed rail carriage seating plans, which can serve as a reference for aircraft; for example, strategies to minimize overall infection risk [9] and short-distance passenger seating arrangements that minimize the number of infected individuals based on virus spatial distribution characteristics [10]. At present, the research on the probability of virus infection among airplane and high-speed train passengers can be mainly divided into these three categories. The first is to combine CFD and the Wells-Riley model to simulate the movement trajectory of droplets containing viruses and calculate the distribution of virus concentration in the cabin [11, 12]. Studies employing this approach always concentrate on the infection being fixed at a specific location or only select a few cabin rows for simulation to improve the model’s accuracy [13, 14], complicating the performance of multiple studies on various passenger layout scenarios within the cabin [15]. The second method uses CO_2_ concentration as an indicator of exhaled gas exposure and predicts the infection risk in unsteady conditions, such as changes in ventilation [16]. However, this method is only applicable when there are no other sources of CO_2_ within the room. The third method uses mathematical models to simulate viral spreading and predict passenger infection risk using indoor ventilation patterns. Zhang and Lin [17] proposed an evaluation method based on virus dilution to assess the spatial and temporal resolution of infection risk in the air. However, the special ventilation situation in aircraft and the effect of nose orientation on virus droplet spreading were not considered.

Here, to solve this problem, a cabin passenger infection risk model based on the Wells–Riley equation was developed by combining the principle of virus dilution and considerations of the special ventilation conditions of the cabin. The model’s validity was verified using measured and surveyed data, and the infection risk associated with different seating arrangement strategies in the cabin was studied.

## 2 Methods

An infectious disease model for studying aircraft cabin scenarios was developed. First, the special environment of the cabin was studied by analyzing the cabin ventilation mode and seat distribution. Then, the infectious disease model was established according to the improved Wells–Riley model.

### 2.1 Aircraft cabin environment

**Fig 1:**
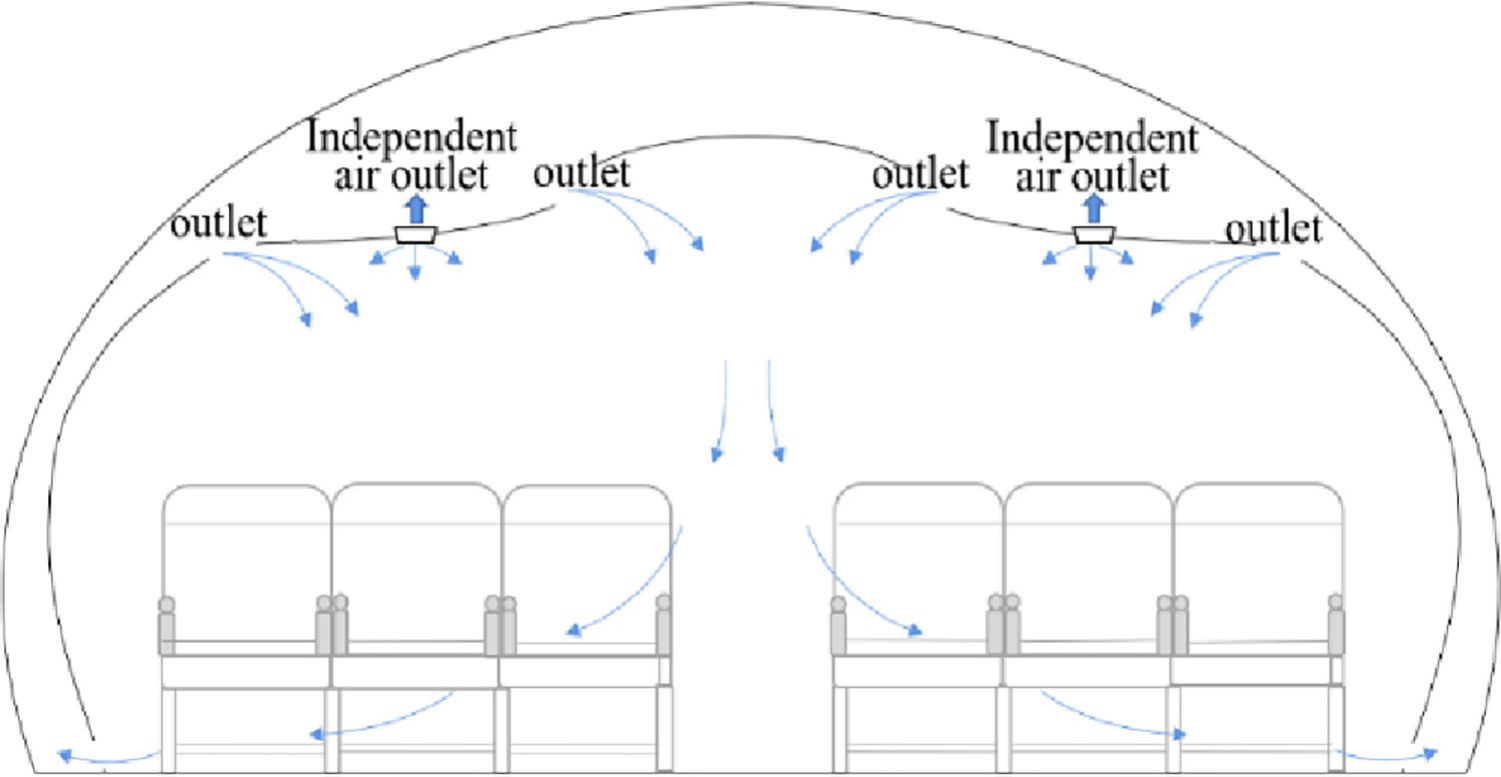
Air circulation in an aircraft cabin(A318/A319/A320/A321).

The ventilation environment and seat distribution of the cabin(A318/A319/A320/A321) are shown in Fig 1. The airplane cabin has specific structural features and real-time air supply patterns that differ from ordinary enclosed spaces [14, 18].

The aircraft air conditioning system refreshes the cabin air every three minutes, much faster than in other indoor environments, and the volume of air available is approximately 80 times that which passengers need to breathe. In modern commercial airliners, the air conditioning system has a recirculation system equipped with high-efficiency particulate air (HEPA) filters that can block 99.97% of bacterial and viral particles. New filter materials also have disinfection and sterilization capabilities; however, transmission can always occur before filtering. Studies have shown that HEPA filters are effective for passengers distant from the infection source but not those in close proximity [18]. The cabin’s Environmental Control System (ECS) mainly creates air circulation in the transverse direction, dispersing aerosols faster in the transverse direction than in the longitudinal direction [13].

In addition, vacant seats can affect the circulation of virus particles [19]. If the seat between the infected and susceptible passengers is unoccupied, droplets can settle on the empty seat surface and be transferred to the next passenger who occupies that seat. Thus, vacant seat strategies influence horizontal virus spreading more than vertical spreading.

### 2.2 The infectious disease model

The Wells–Riley formula was improved to establish an infectious disease model as previously described [20–22]. The Wells–Riley formula is derived as follows:

$$P=\frac{C}{S}=1-\exp(-Ir)=1-\exp(-Ipqt/Q)$$ (1)

Where *P* is the probability of infection, *C* is the number of infection cases, *S* is the number of susceptibles, *I* is the number of infectors, *r* is the effective contact rate, and *p* is the pulmonary ventilation rate of a person (*m*^3^/*h*). When people are sitting or participating in light activities indoors, p = 0.3 m3/h [23], and *q* is the quanta generation rate (*quanta*/*h*). To date, no q values have been obtained directly for SARS-CoV-2; Hui Dai [24] collected known Q and R_0_ (the average number of infectious individuals created by a single infector in a susceptible population [25]) values for other airborne infectious diseases to obtain a reasonable q value. *t* is the exposure time interval (*m*^3^/*h*), and *Q* is the room ventilation rate (*m*^3^/*h*).

Regarding Formula (1), even though it can be used to predict outbreaks of various airborne diseases, the impact of defensive measures, such as wearing masks, on the probability of infection is not considered. Thus, the mask permeability coefficient (*θ*) is introduced into the formula [26, 27] to take into account the impact of masks and particle filtration on viral spreading, yielding the following equation:

$$P=\frac{C}{S}=1-\exp(-Ipqt\theta /(Q_{\eta }+Q_{r}\mu_{r}))$$ (2)

Where *Q*_*r*_ is the flow rate to the filter, *Q*_*η*_ is the fresh air ventilation rate and *μ*_*r*_ is the filtration efficiency.

The air supplied on board an aircraft is half HEPA-filtered and half fresh air, so yielding the following equation:

$$Q_{r}=Q_{\eta }=\frac{1}{2}Q=\frac{1}{2}V\kappa$$ (3)

Where *V* is the cabin volume, *κ* is the cabin air exchange rate.

*θ* is the mask permeability coefficient and ranges from 0 to 1; 0 represents complete isolation of the pathogen by the mask, while the filtration efficiency of a standard surgical mask for an aerosol containing viruses is approximately 60% [28]. Considering the potential for some passengers to experience air leakage when wearing masks, *θ* can be set at 50% to eliminate the impact of this factor [10].

There is a significantly higher risk of infection from close-range droplet transmission and airborne transmission than from far-range airborne transmission [18, 29], and the probability of infection is related to the distance between the infected and susceptible individual. Thus, we assume the concentration distribution of the virus in the cabin follows a Gaussian distribution and other modifications should be made as follows:

1. The number of passengers in the cabin and the total level of viral particles exhaled by passengers are assumed to be constant.
2. The death rate of the virus during its spread can be ignored.
3. The probability of infection can be accumulated.
4. Passenger movement and cabin service are not considered.
5. Each seat and aisle in the cabin is regarded as an individual space, and the virus concentration remains the same within this space.

Based on the above assumptions, the average virus concentration within the individual space where the infected individual *i* is located can be expressed as:

$$\omega_{i}=\frac{q_{i}}{(Q_{\eta }+Q_{r}\mu_{r})/num}$$ (4)

Where *num* represents the number of individual spaces and (*Q*_*η*_+*Q*_*r*_*μ*_*r*_)/*num* is the ventilation rate per space, also referred to as the individual ventilation rate.

The virus concentration follows a Gaussian distribution; the average virus concentration within the individual space where susceptible individual *j* is located and the effective contact rate [10] between susceptible individual *j* and infected individual *i* in the cabin can be expressed as:

$$\omega_{j}=\omega_{i}\exp[-\frac{{\left(d_{ij}-\mu_{i}\right)}^{2}}{2\delta_{i}^{2}}]$$ (5)

$$r_{ij}=\frac{p_{j}\omega_{i}t\theta }{\sqrt{2\pi }\delta_{i}}\exp[-\frac{{\left(d_{ij}-\mu_{i}\right)}^{2}}{2\delta_{i}^{2}}]$$ (6)

Where *ω*_*j*_ is the average virus concentration within the individual space of susceptible individual *j*, *r*_*ij*_ is the effective contact rate, *p*_*i*_ is the pulmonary ventilation rate of susceptible individual *j*, *q*_*i*_ is the quantum generation rate by infected individual *i*, *δ*_*i*_ is the Gaussian distribution parameters related to the virus, *d*_*ij*_ is the distance between infected individual *i* and susceptible individual *j*, and *μ*_*i*_ is the Gaussian distribution parameters related to the virus.

Compared to the traditional Wells–Riley model, the model described above provides a more detailed and refined risk assessment strategy considering the efficacy of filters. Calculating the infection risk probability of each susceptible individual based on their distance from the infected individuals has significant implications for studies on the distribution of empty seats when the occupancy rate is less than 100%.

However, the horizontal and longitudinal spread intensity of the virus in the model was consistent, and the aircraft cabin’s special ventilation environment was not considered. Virus transmission in the cabin primarily occurs through the air, with the diffusion of virus concentration in both transverse and longitudinal directions, following Gaussian distribution but with different amplitudes, and the virus is more concentrated in the transverse than longitudinal direction. In the cabin, the virus spreads from an area of high concentration to an area of low concentration.

Suppose there are n infected individuals and their seats are (*x*_1_, *y*_1_)、(*x*_2_, *y*_2_)……(*x*_*n*_, *y*_*n*_) in an aircraft cabin, where *x* is the transverse direction and *y* is the longitudinal direction. The effective contact rate of the other passengers can be represented as follows:

$$r(x,y)=\sum_{i=1}^{n}p_{j}\omega_{i}t\theta \beta \frac{1}{\sqrt{2\pi }\delta_{x}}\exp[-\frac{{\left(x_{i}-x\right)}^{2}}{2\delta_{x}^{2}}]\frac{1}{\sqrt{2\pi }\delta_{y}}\exp[-\frac{{\left(y_{i}-y\right)}^{2}}{2\delta_{y}^{2}}]$$ (7)

Where *δ*_*x*_ is the related transverse Gaussian distribution parameters regarding the virus, and *δ*_*y*_ is the Gaussian distribution parameters related to the virus in the longitudinal direction.

Parameter *β* was added to describe the difference in longitudinal flow. When the susceptible person is in front of the infected person, *β* is between 0 and 1; when the susceptible person is seated behind the infected person, the value of *β* is 1 [19].

Given the effect of occupied and unoccupied seats on airflow in the cabin, Formula (8) can be modified as follows:

$$\frac{r(x,y)}{r^{,}(x,y)}={\left(\frac{v}{v-v_{row}}\right)}^{n}$$ (8)

Where *r*(*x*, *y*) represents the effective exposure rate of the susceptible individual at (*x*, *y*) without considering the situation where the seat is unoccupied, *v* is the volume of a seat in the cabin, *n* is the he number of empty seats horizontally between the susceptible and infected person, and *v*_*row*_ is the volume of space occupied by a passenger.

Considering every possible factor, the infection probability *P*_*j*_ for susceptible *j* under the influence of n infected individuals can be obtained by the Wells equation:

$$P_{j}=1-\exp(-\sum_{i=1}^{n}r_{ij}’)$$ (9)

Where *n* is the number of infected individuals and *r*_*ij*_’ represents the effective exposure rate of the susceptible individual *j* with considering the situation where the seat is unoccupied.

### 2.3 Model validation

The passenger information from two flights was used to verify the model’s accuracy, as described in detail below.

#### 2.3.1 Case 1

On March 23, 2020, the SARS-CoV-2 spread within the cabin of an airplane traveling from B County airport to Okinawa [30]. An investigation was conducted on the airplane and the passengers and flight crew on board. The plane was a Boeing 737–800 with 177 economy-class seats and an air recirculation system with high-efficiency particulate air (HEPA) filters. There were 148 people on board, including 7 index cases, 139 other passengers, 4 flight attendants, and 2 pilots. The pilots did not share the cabin with passengers. After the flight, 8 confirmed cases and 8 suspected cases were identified. The model parameters used are shown in Table 1 [29, 31, 32].

**Table 1:** Model parameters in Case 1.

*q* (quanta·h^-1^)	100
*p* (m^3^·h^-1^)	0.49
*t* (h)	3
*Q*_*η*_ (m^3^·h^-1^)	3454.5
*Q*_*r*_ (m^3^·h^-1^)	3454.5
*μ*_*r*_	99.7%
*θ*	0.5
*β*	0.35
Seat width (m)	0.482
Seat pitch (m)	0.726
Aisle width (m)	0.51
num	210

**Fig 2:**
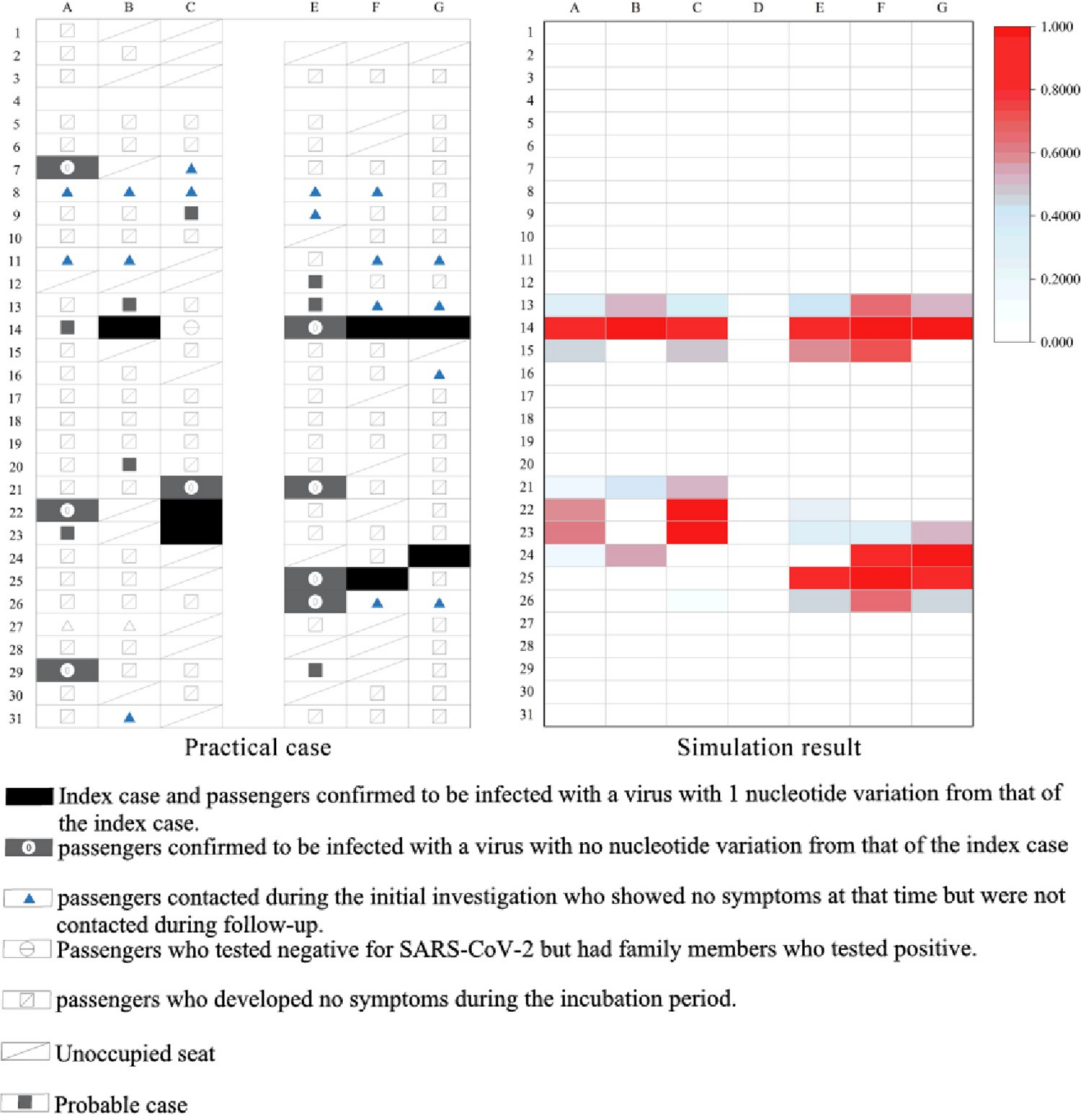
Cabin infection pattern in Case 1.

In Fig 2, grey square and 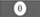 along with the index cases are classified as high risk for infection; the remaining are classified as low or medium risk in the practical case. In the simulation result, we define passengers (0.5≤*P*_*j*_≤1) as high-risk for infection. The infection probability of each location corresponds to each individual situation.

However, whether the passenger in this seat is infected depends on their immunity. As shown in Table 2, despite this discrepancy in cases, the model simulated the case with a precision rate of 89.3% (134/150). It should be noted that the poor match in Fig 2 (A7:C9) may be because one of the infected people was not detected at the beginning of the flight.

**Table 2:** Case 1 data.

Scene type	Index cases	High risk		Others	
		Total	Match	Total	Match
Practical case	7	16	7	128	121
Simulation result	7	15		129	

#### 2.3.2 Case 2

On September 28, 2020, the new coronavirus spread within the cabin on an airplane traveling from Dubai, UAE, to Auckland, New Zealand. The case only displays the distribution of passengers in rows 24–30. Through genome sequencing, scientists discovered that four passengers on board were infected by one of two other passengers. The model parameters used are shown in Table 3 [29, 31, 32].

**Table 3:** Model parameters in Case 2.

*q*(quanta·h^-1^)	100
*p*(m^3^·h^-1^)	0.49
*t*(h)	18
*Q*_*η*_ (m^3^·h^-1^)	11093.9
*Q*_*r*_ (m^3^·h^-1^)	11093.9
*μ*_*r*_	99.7%
*θ*	0.5
*β*	0.35
Seat width (m)	0.482
Seat pitch (m)	0.726
Aisle width (m)	0.51
num	390

**Fig 3:**
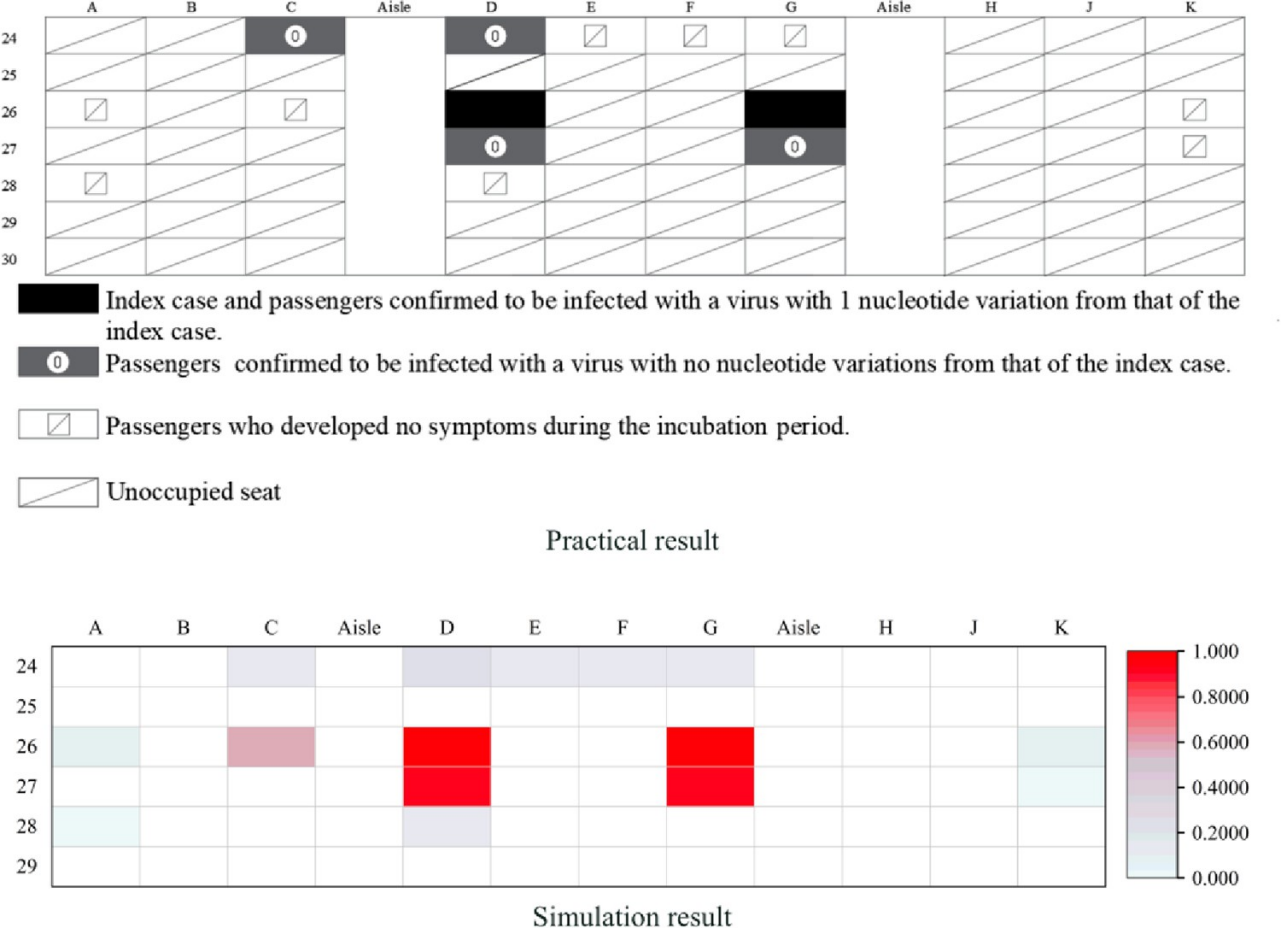
Details of the cabin infection in Case 2.

We classified 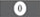 and the index case as high-risk for infection (Fig 3) in the practical case, and those in the other seats as low and medium risk. We defined passengers (0.5≤*P*_*j*_≤1) as high-risk for infection in the simulation result (Fig 3).

As shown in Table 4, the model simulated the case with a precision rate of 86.7% (13/15). It should be noted that the mismatch in passengers (C,24) shown in Fig 3 may have arisen from passenger movement.

**Table 4:** Case 2 data.

Scene type	Index cases	High risk		Others	
		Total	Match	Total	Match
Practical case	2	4	2	9	9
Simulation result	2	2		11	

## 3 Results

The verified model described above was used to study the effects of passenger load factor and empty seat position on the probability of passenger infection. A reasonable seating arrangement is then proposed.

### 3.1 Evaluation of passenger infection risk based on the passenger load factor

**Fig 4:**
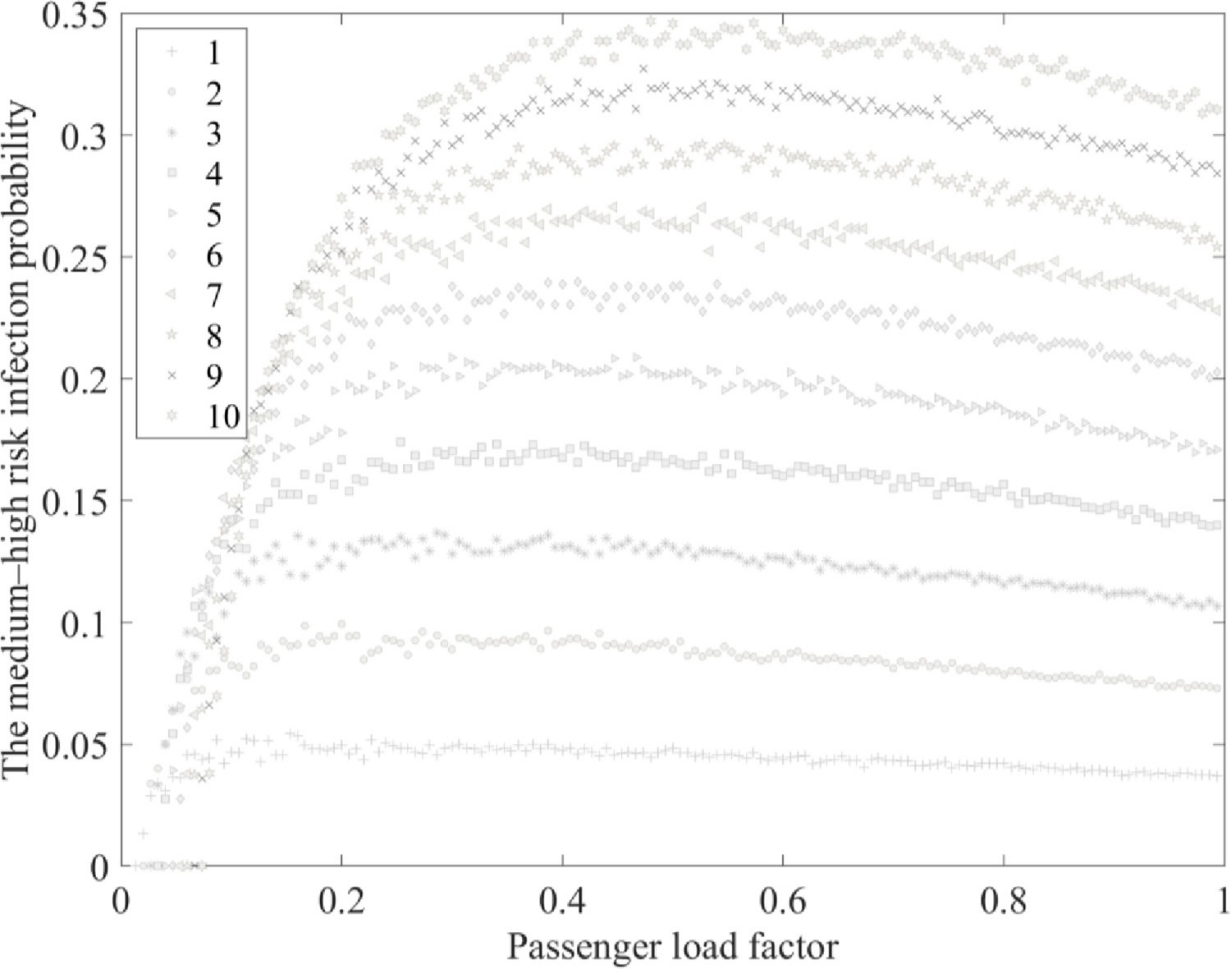
The relationship between load factor and the medium–high risk infection probability.

**Fig 5:**
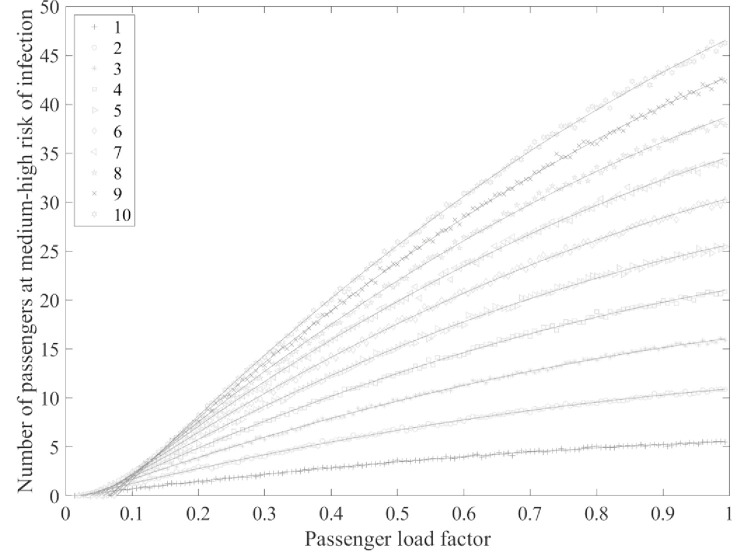
The relationship between passenger load factor and the number of passengers at medium–high risk of infection.

We assessed the trend in the number of passengers at medium–high risk for infection in the cabin as the passenger load factor changed with varying initial numbers of infected passengers. The average value was taken from one thousand calculations for each scenario, and the results are shown in Figs 4 and 5.

**Fig 6:**
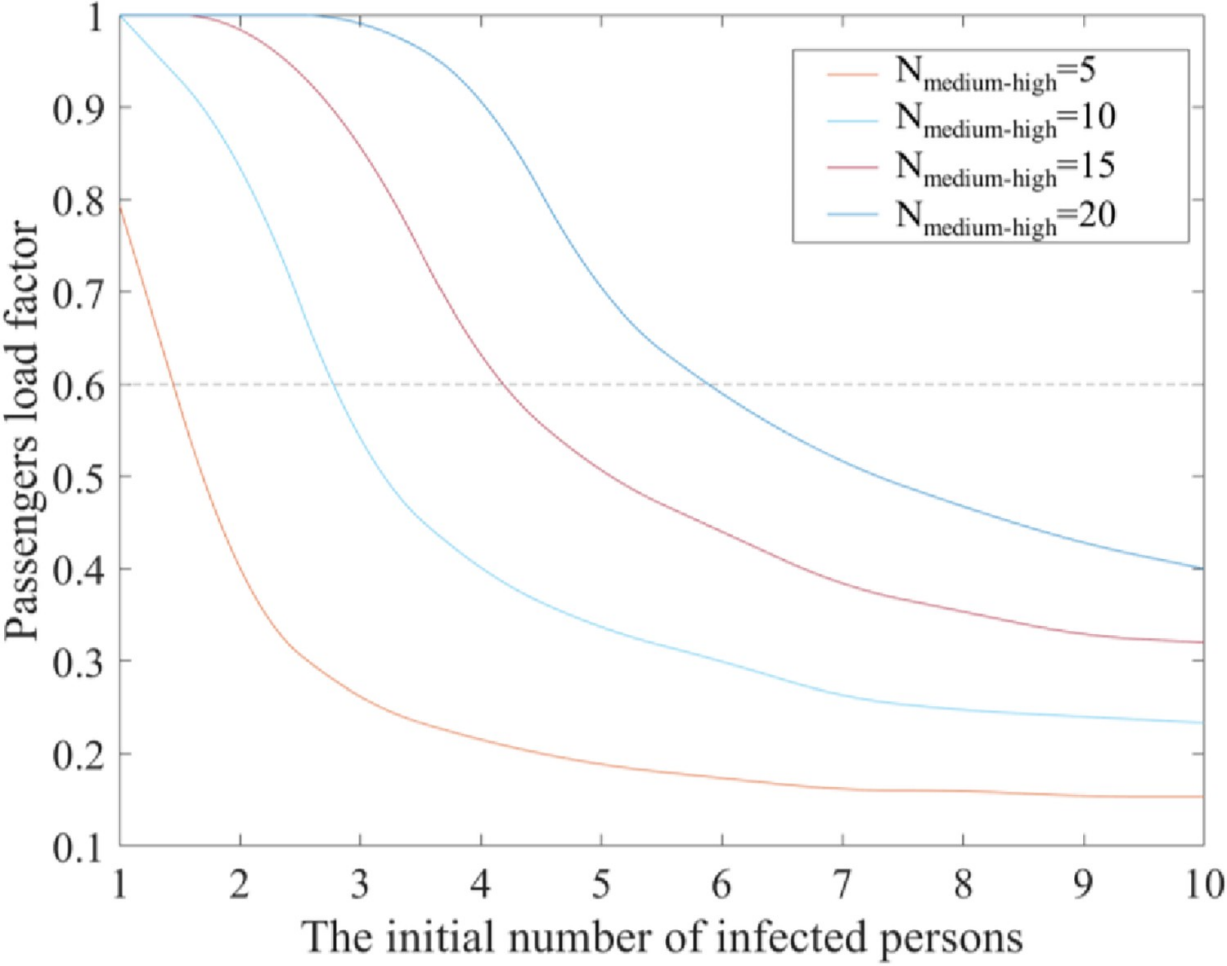
The relationship between the initial number of infected passengers and the passenger load factor.

**Fig 7:**
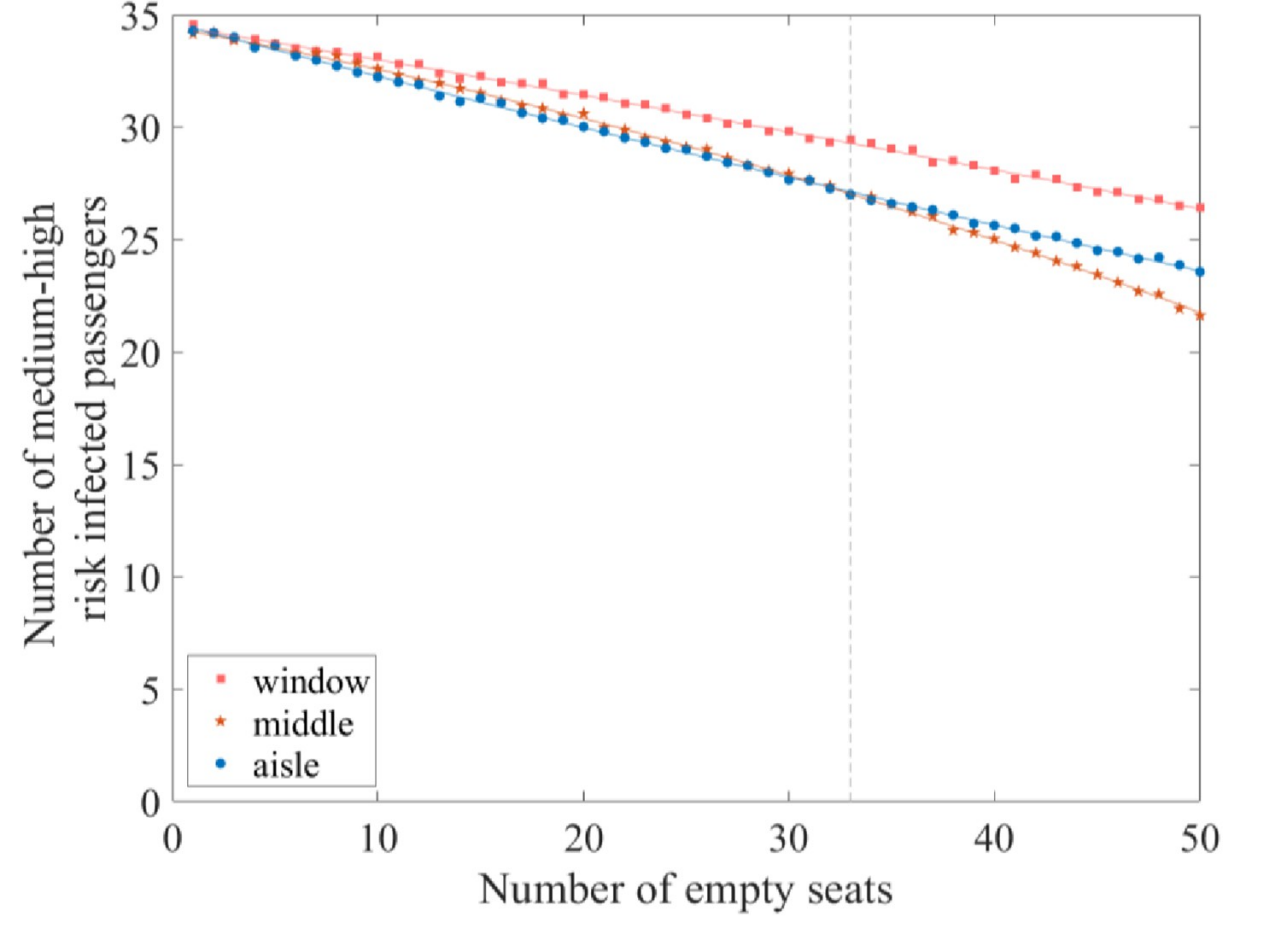
The relationship between the number of vacant seats and the number of passengers at medium–high risk for infection.

**Fig 8:**
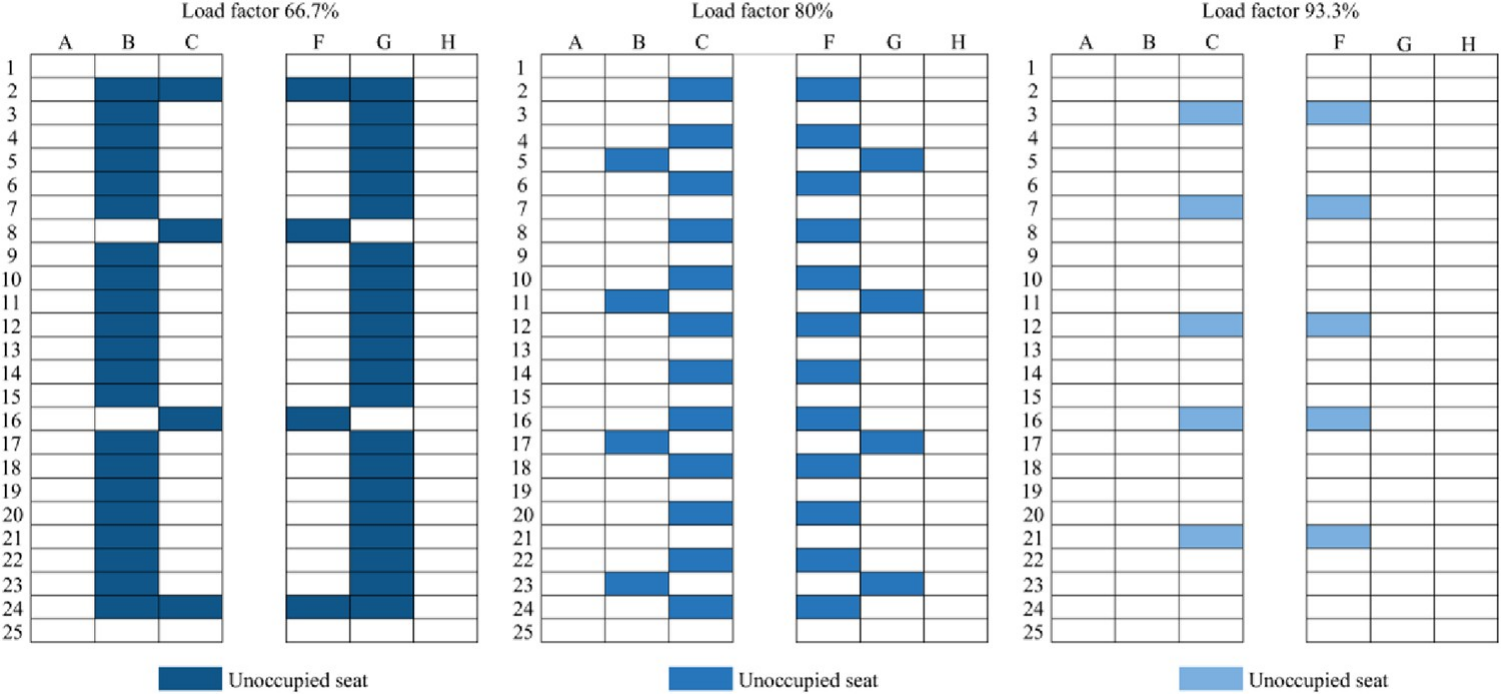
Load factors achieved through the recommended seating arrangement.

**Fig 9:**
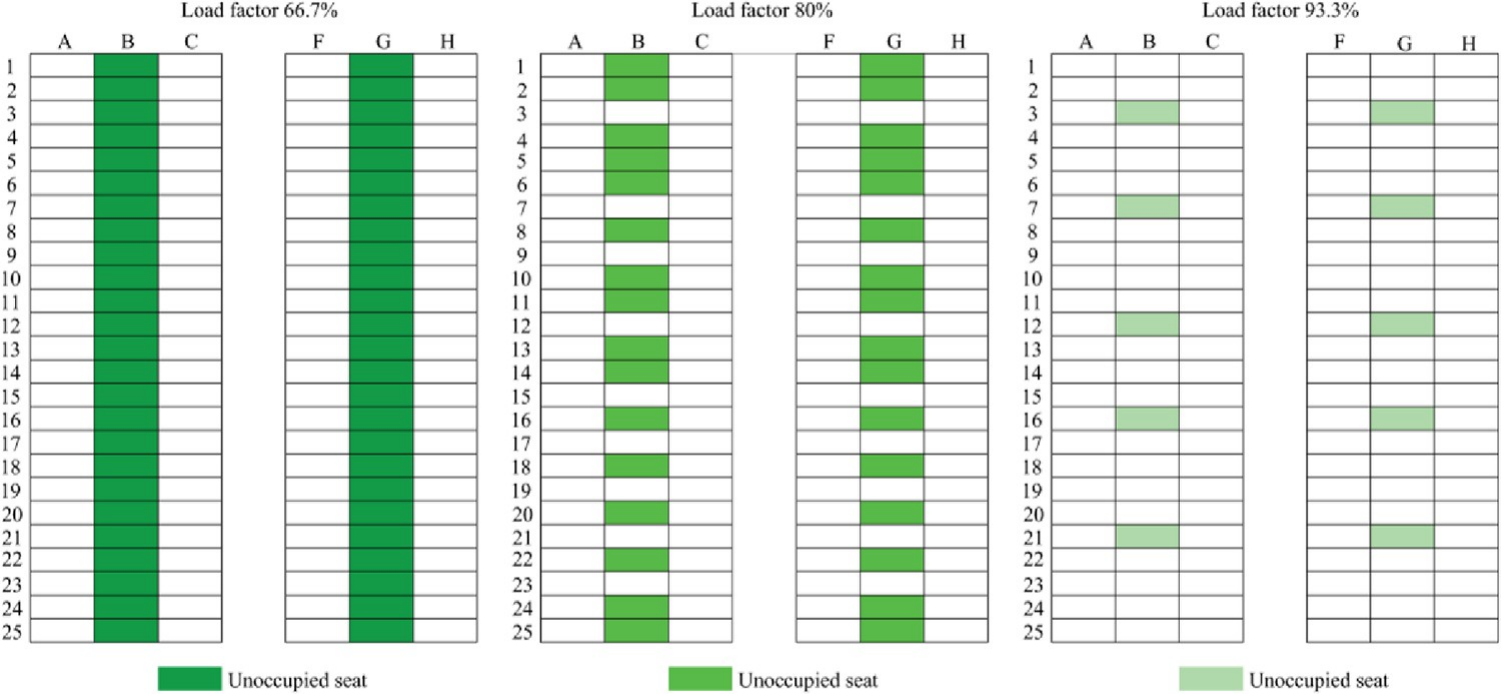
Load factors achieved through leaving some or all middle seats vacant.

**Fig 10:**
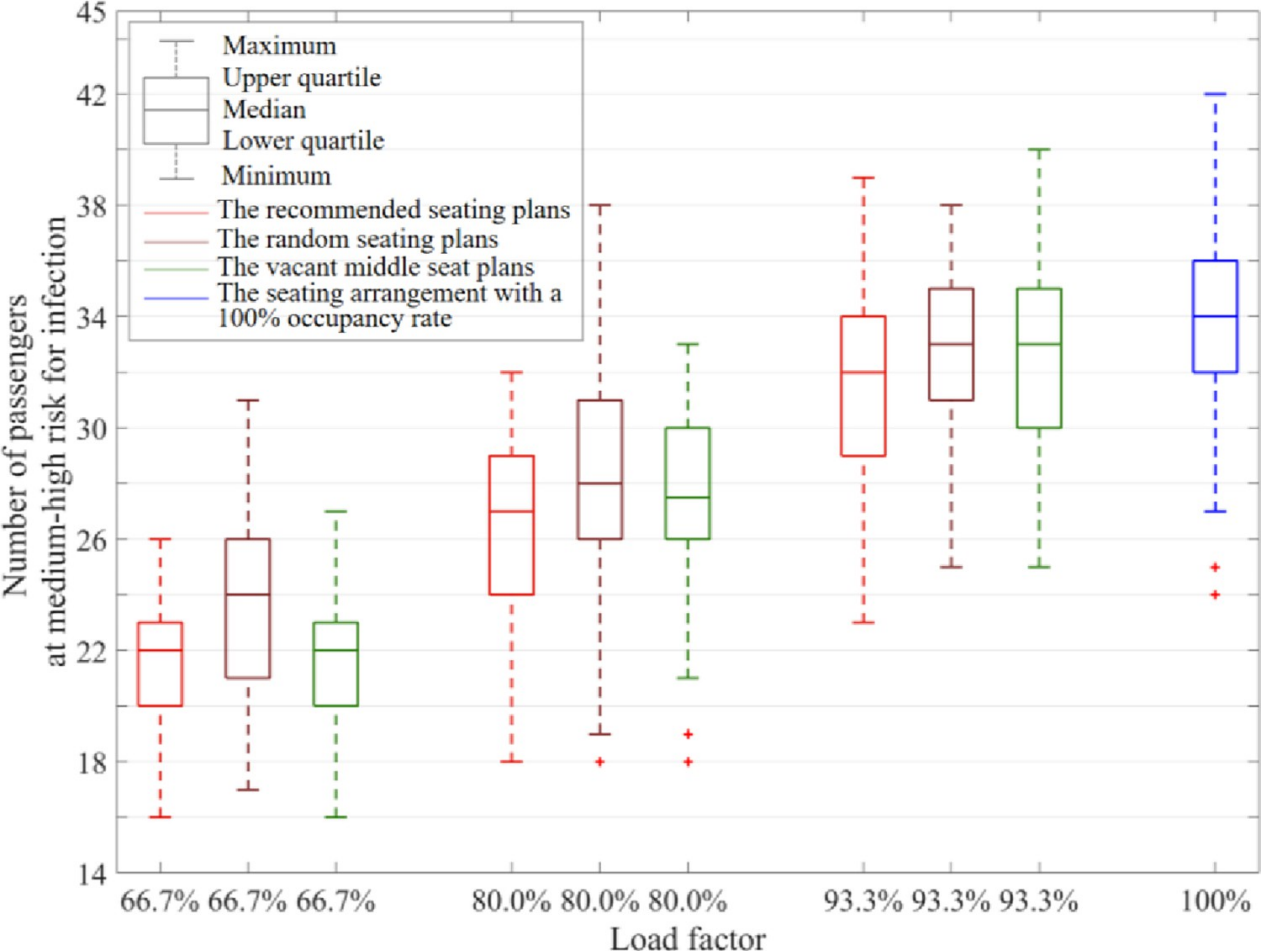
Comparison of recommended, random seating and VMS arrangement plans.

It is thought that if the infection probability is higher than 0.2, individual differences among passengers may lead to eventual infection. Therefore, the number of passengers at medium–high risk was analyzed in the main study. Figs 4 and 5, 1–10 show the number of people initially infected and trend lines demonstrating the relationship between the passenger load factor and the number of passengers at medium–high risk of infection ($0.2\leq P_{j}\leq 1$).

As the passenger load factor increases, the number passengers at medium–high risk for infection gradually increases, the medium–high risk infection probability first increases and then decreases, and the passenger load factor at the peak of the medium–high risk infection probability differs depending on initial infection numbers. The greater the initial number of infections is, the larger the passenger load factor is at the peak.

The passenger load factor is the key to the profitability of the air transport industry. This article assesses the maximum passenger load factor that can be used to control the final number of infected passengers when the initial number of infected people is different.

The relationship between the number of passengers at medium–high risk for infection and the maximum passenger load factor was studied with differing initial numbers of infected passengers, as shown in Fig 6.

As shown in Fig 6, *N*_*medium–high*_ represents the number of passengers at medium–high risk for infection. The black dotted line represents a passenger load factor of 60%. It is demonstrated that an aircraft can recoup the cost of one flight if its passenger load factor reaches 60%.

If an aircraft can recoup the cost, when the final number of infected persons is controlled at 5, the initial number of infected passengers must be equal to 1 or less than 1. When the final number of infected persons is controlled at 10, the initial number of infected passengers must not exceed 3. When the final number of infected persons is controlled at 15, the initial number of infected passengers must not exceed 5. When the final number of infected persons is controlled at 20, the initial number of infected passengers must not exceed 6. The flight seating arrangement plan can be adjusted based on the local epidemic situation to control the number of infected individuals at the origin of the flight.

### 3.2 Analysis of the effect of seating arrangement on passenger infection risk

This section examines the impacts of empty seat positions on the probability of infection among passengers. The cabin seats can be divided into three categories according to their proximity to the windows and the aisle: window, aisle, and middle. Numbers of empty seats ranging from 1 to 50 were assessed with an initial infected population of 7 (as shown in the practical case in section 2.3) to investigate the relationship between the empty seat location and the probability of passenger infection. Three empty seat arrangements, that is, window seats, middle seats, and aisle seats, were evaluated in terms of the number of passengers at medium–high risk for infection.

As shown in Fig 7, when the number of vacant seats is lower than 33, the arrangement of vacant seats in the aisle column reduces the number of passengers at medium–high risk for infection. However, when the number of vacant seats is greater than 33 and lower than 50, the arrangement of vacant seats in the middle column reduces the number of passengers at medium–high risk for infection. When the number of vacant seats is 50, and all are placed in the middle column, the number of passengers at medium–high risk for infection is the lowest, decreased by 37.47%((34.59–21.63)/34.59) in comparison with the scenario leading to the maximum number of passengers at medium–high risk for infection.

### 3.3 Analysis of the effect of seating arrangement on passenger infection risk

Seating arrangement plans (Fig 8) were designed for three load factors (66.7%, 80.0%, and 93.3%) based on the associations between seating patterns and the ratio of infected travelers versus the number of passengers at medium–high risk of infection. And the vacant middle seat scenarios (VMS) for three load factors (66.7%, 80.0%, and 93.3%) also were shown in Fig 9. It should be noted that, unlike the usage of VMS in Dietrich [8] and Bennett [7], the VMS refers to leaving the vacant seats in the middle column due to the uncertainty of the infected person’s location.

The positions of the 7 initial infectors were randomly allocated, and the numbers of travelers at a medium–high risk for infection after a 3-hour journey were compared across the recommended plans、vacant middle seat plans (VMS) and random seating distribution plans per load factor. The simulation result summary is shown in Fig 10.

In Fig 10, the box plots represent the number of travelers at a medium–high risk for infection under the recommended seating plans、the random seating plans and the vacant middle seat plans respectively at passenger load factors of 66.7%, 80%, 93.3% and 100%. And The relevant data is recorded in Table 5.

**Table 5:** Comparison of recommended and random seating arrangement plans.

Load factor	Seating plans	Maximum	Upper quartile	Median	Mean value	Lower quartile	Minimum
66.7%	Recommended	26	23	22	21.48	20	16
	Random	31	26	24	23.62	21	17
	VMS	27	23	22	21.82	20	16
80.0%	Recommended	33	29	27	26.68	24	18
	Random	38	31	28	27.96	26	19
	VMS	33	30	27.5	27.38	26	21
93.3%	Recommended	39	34	32	31.60	29	23
	Random	38	35	33	32.62	31	25
	VMS	40	35	33	32.88	30	25
100%		42	36	34	33.84	32	27

According to Fig 10 and Table 5, compared to the random seating arrangement, both VMS and the recommended approach proposed in this paper effectively reduce the risk of passenger infection at different occupancy rates. VMS and the recommended seating plans reduce the numbers by 35.52% and 36.52% respectively in comparison with the average number of travelers at a medium–high risk for infection at 100% occupancy rate. When the occupancy rate is 66.7%, the recommended approach and VMS show similar performance. However, as the occupancy rate increases, the recommended approach demonstrates greater advantages. When the occupancy rate is 93.3%, the recommended approach reduces 3.89% more than VMS.

## 4 Discussion

Due to the multiple variants of SARS-CoV-2 and the differences in research environments, variations exist in q values, which are crucial parameters determining the model’s results. Here, the q values and their impact on the number of infections in the medium to high-risk population is discussed. The study findings provide a reference for assessing infection risk with different q values. Some airlines have suspended cabin services due to the widespread transmission of the virus, and passengers have voluntarily reduced their cabin activities; thus, in this study, it was assumed that passengers do not move within the cabin. However, passenger movement will inevitably affect the infection situation. Thus, the impact of infected individuals’ movement is also discussed here.

### 4.1 Analysis of the impact of q values on the number of passengers at medium–high risk for infection

**Fig 11:**
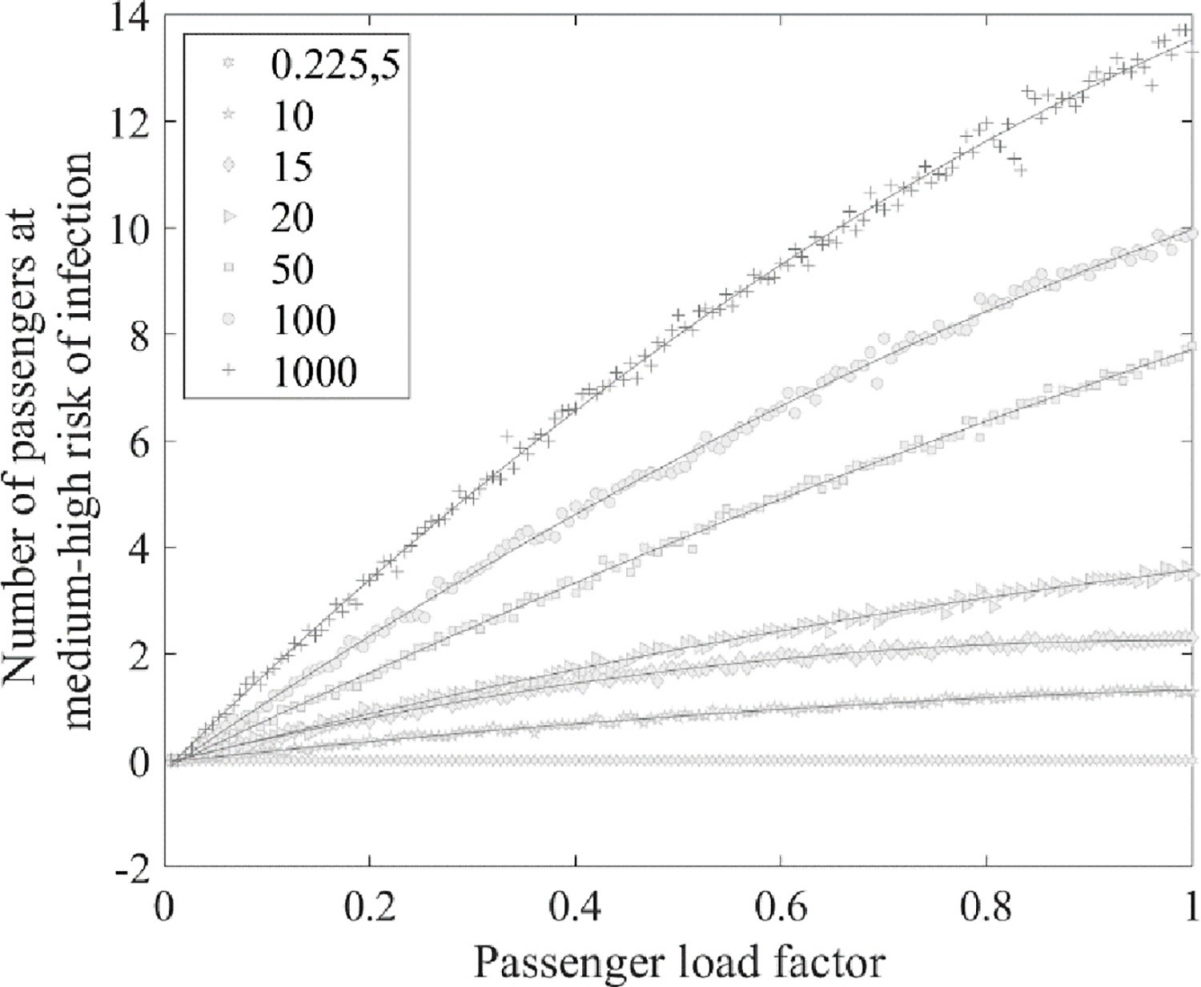
The relationship between the number of passengers at medium–high risk for infection and the passenger occupancy rates under different q values.

The q values vary due to the multiple variants of SARS-CoV-2 and the differences in research environments. Hota [33] calculated a q value of 0.225 for a hospital ward using the established airborne transmission model. Dai and Zhao et al. [24] determined that the q value for SARS-CoV-2 would range from 14–48 quanta/h based on the fitted correlation between the q value and the basic reproduction number (R0). Miller and Nazaroff et al. [34] estimated the q value of 970 ± 390 quanta/h in an analysis of a super-spreading choir event. Combining exhalation activities and activity levels, Buonanno [35] measured the q values in four scenarios: (i) oral breathing during resting; (ii) oral breathing during heavy activity; (ii) speaking during light activity; and (iv) singing (or loudly speaking) during light activity. The respective q values obtained were 0.37, 2.5, 5.0, and 32 quanta/h. Buonanno [31] also calculated that the q value of an asymptomatic infectious subject performing vocalization during light activity would exceed 100 quanta/h due to the viral load in the mouth, the type of respiratory activity, respiratory physiological parameters, and the activity level. By compiling the literature on reported COVID-19 events, Wang [36] considered three quantum generation rates, 100 quanta/h, 20 quanta/h, and 5.0 quanta/h, representing nominally severe, moderate, and mild scenarios, respectively. In this study, q values of 0.225, 5, 10, 20, 50, 100, and 1000 quanta/h were selected. Simulations were conducted with an infected individual and varying passenger occupancy rates from 0 to 100% to investigate the relationship between the number of passengers at medium–high risk for infection and the passenger occupancy rates under different q values,

As shown in Fig 11, the q value significantly influences the final number of infections. As the value of q increases, the rate at which the number of infections increases with the passenger occupancy rate also increases.

### 4.2 The impact of passenger movement on the passengers’ infection situation

Changes in virus concentration within the cabin when infected individuals move were assessed using a dynamic infection risk assessment model.

**Fig 12:**
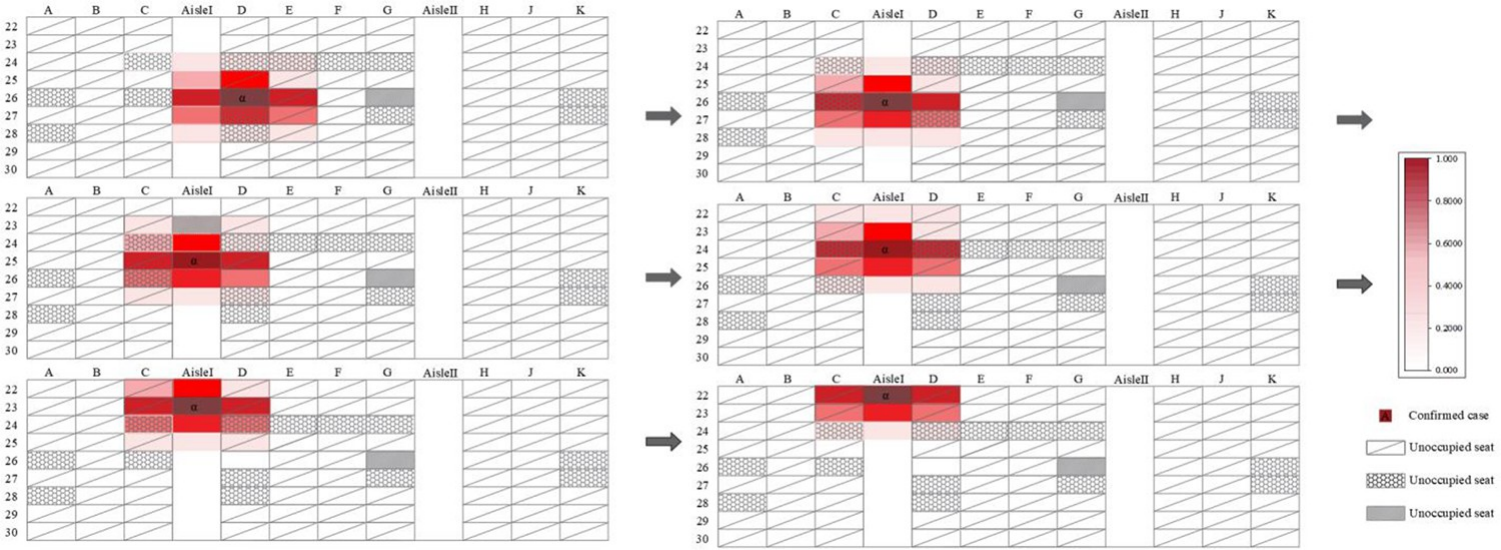
Walking route of infected individual α.

The following assumptions were made:

1. Assuming that the infected individual *α* chooses the nearest restroom (located at Row 18), the walking route will be as shown in Fig 12: (Aisle I, 26)-(Aisle I, 18); the passenger will return after 3 minutes following the route (Aisle I, 18)- (Aisle I, 26).
2. The aircraft cabin is divided into corresponding individual cells based on seat and aisle arrangements. The virus concentration in each cell is related to the distance between the cell and the infected individual, and the virus concentration remains constant within each cell.

Based on the above assumptions, as infected individual *α* moves, the virus concentration in each cell changes. The virus concentration at (*x*_*i*_, *y*_*i*_) can be calculated as follows when the infected individual *α* is located at (Aisle I, n):

$$\omega_{x_{i},y_{i}}(AsileI,n)=\omega_{\alpha }\frac{1}{\sqrt{2\pi }\delta_{x}}\exp[-\frac{{\left(x_{i}-x\right)}^{2}}{2\delta_{x}^{2}}]\frac{1}{\sqrt{2\pi }\delta_{y}}\exp[-\frac{{\left(y_{i}-y\right)}^{2}}{2\delta_{y}^{2}}]$$ (10)

Where *ω*_*α*_ represents the average virus concentration within the cell of the infected individual *α*, *δ*_*x*_ represents the related transverse Gaussian distribution parameters regarding the virus, *δ*_*y*_ represents the Gaussian distribution parameters related to the virus in the longitudinal direction, (*x*_*i*_, *y*_*i*_) represents the location of susceptible individual *i*, and (*x*, *y*) represents the location of infected individual *α*.

Assuming that the passenger takes *t*_*walk*_ to pass through each cell, the effective contact rate between the susceptible individual *i* and the infected individual *α* when *α* travels to the restroom can be expressed as follows:

$$r(AsileI,18-AsileI,26)=\sum_{n=18}^{26}p_{j}\omega_{x_{i},y_{i}}(AsileI,n)t_{walk}\theta \beta$$ (11)

Assuming that infected individual *α* returns to his or her original seat after 3 minutes following the route (Aisle I, 18)- (Aisle I, 26), the effective contact rate during this 3-minute can be expressed as follows:

$$r(wait_{3\min})=\sum_{n=18}^{26}\frac{3p_{j}\omega_{x_{i},y_{i}}(AsileI,n)\theta \beta }{60}$$ (12)

The air in the cabin is replaced approximately 20 times per hour, with half of the air being refreshed each time. The effective contact rate when the infected individual *α* is returning to (D, 26) can be expressed as follows:

$$r(AsileI,26-AsileI,18)=\frac{3*\sum_{n=18}^{26}p_{j}\omega_{x_{i},y_{i}}(AsileI,n)t_{walk}\theta \beta }{2}$$ (13)

When the infected person *α* returns to (D, 26), his or her movement continues to have residual effects on the surrounding positions, especially the seats on both sides of the aisle. When the virus concentration at susceptible individual *i* returns to the same level as *ω*_*st*_ (the virus concentration when the infected person *α* is stationary at (D, 26)), the effective contact rate can be expressed as follows:

If $\frac{3*\sum_{n=18}^{26}\omega_{x_{i},y_{i}}(AsileI,n)}{2*2^{n}}>\omega_{st}$ and $\frac{3*\sum_{n=18}^{26}\omega_{x_{i},y_{i}}(AsileI,n)}{2*2^{n+1}}<\omega_{st}$

$$\begin{array}{c} r(re\mathrm{cov}er)=\frac{3*\sum_{n=18}^{26}p_{j}\omega_{x_{i},y_{i}}(AsileI,n)(3-t_{walk})\theta \beta }{2^{1}}+\frac{3*3*\sum_{n=18}^{26}p_{j}\omega_{x_{i},y_{i}}(AsileI,n)\theta \beta }{60*2^{2}}+\\ \ldots \ldots +\frac{3*3*\sum_{n=18}^{26}p_{j}\omega_{x_{i},y_{i}}(AsileI,n)\theta \beta }{60*2^{n}} \end{array}$$ (14)

The effective contact rate between susceptible individual *i* and infected person *α* during the movement of infected person *α* can be expressed as follows:

$$r_{move}=r(AsileI,18-AsileI,26)+r(wait_{3\min})+r(AsileI,26-AsileI,18)+r(re\mathrm{cov}er)$$ (15)

Based on Formula (15) and Formula (7), the effective contact rate between susceptible individual *i* and infected person *α* throughout the journey can be expressed as follows:

$$r(x_{i},y_{i})=\sum_{i=1}^{n}p_{j}\omega_{i}(t‐18t_{walk}-3/60)\theta \beta \frac{1}{\sqrt{2\pi }\delta_{x}}\exp[-\frac{{\left(x_{i}-x\right)}^{2}}{2\delta_{x}^{2}}]\frac{1}{\sqrt{2\pi }\delta_{y}}\exp[-\frac{{\left(y_{i}-y\right)}^{2}}{2\delta_{y}^{2}}]+r_{move}$$ (16)

**Fig 13:**
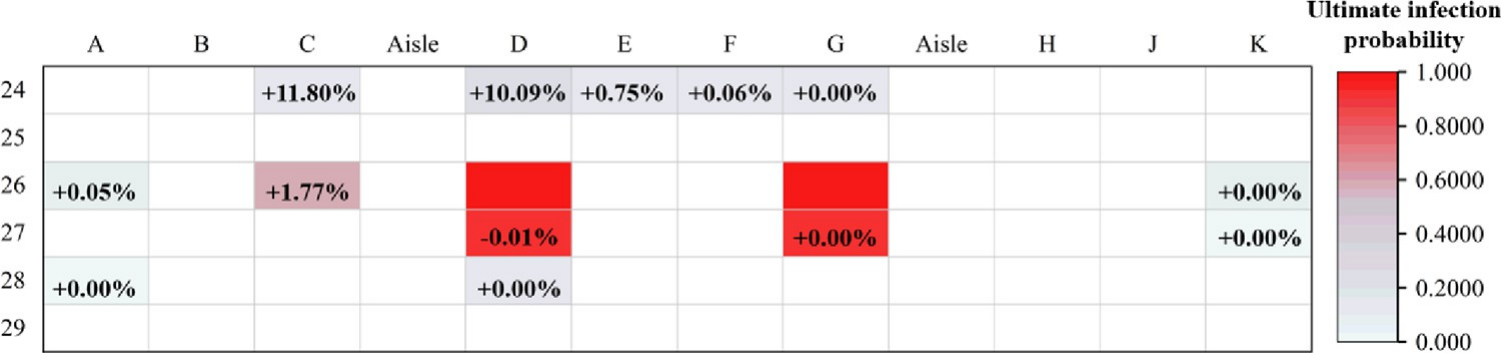
Verification of Case 2 considering passenger movement.

The infection risk for passengers in Case 2 was next evaluated by combining the dynamic infection risk assessment model with the static infection risk assessment model mentioned earlier. When the infected person was walking, the q value was chosen to double the original. The simulation results are shown in Fig 13.

As shown in Fig 13, the number of passengers at medium–high risk for infection in the cabin increases when considering the movement of infected individuals. The accuracy of the model improved to 93.3% (14/15). Specifically, the infection probabilities of susceptible individuals located in the aisle (C, 24) and (D, 24) increased by 11.80% and 10.09%, respectively. The infection probabilities of susceptible individuals in the middle seats (E, 24), (F, 24), and (G, 24) increased by 0.75%, 0.06%, and 0.00%, respectively. Thus, the movement of infected individuals significantly impacts the infection probability of passengers on both sides of the aisle. The impact on other passengers depends on the distance between each passenger and the moving infected individuals. Therefore, it is recommended to minimize passenger movement during an outbreak.

## 5 Conclusions

This study evaluated the risk of passenger infection with SARS-CoV-2 based on the Wells–Riley formula. The main conclusions are as follows:

1. Seating capacity significantly impacts the final passenger infection outcome. As the passenger load factor increases, the number of infected people gradually increases, and the infection probability first increases and then decreases on a per passenger basis. When the outbreak situation of the departure city is clear, the optimal passenger load factor can be determined to control the number of infected people.
2. When the number of vacant seats is lower than 33, the arrangement of vacant seats in the aisle column reduces the number of passengers at medium–high risk for infection. However, when the number of vacant seats is greater than 33 and lower than 50, the arrangement of vacant seats in the middle column further reduces the number of passengers at medium–high risk for infection. And leaving empty seats in middle of the aisle can effectively reduce passenger infection risk by 37.47% in comparison with that observed in the highest infection risk scenario.
3. Compared to the random seating arrangement, both VMS and the recommended seating plan can effectively reduce the probability of passenger infection by 35.52% and 36.52% respectively. The recommended approach performs similarly at 66.67% occupancy rate in comparison with the probability observed with VMS. However, as the occupancy rate increases, the recommended approach can reduce a higher probability of infection, approximately 3.89%.
